# Improved Speech Recognition in Adults With Conductive or Mixed Hearing Loss Using a Direct-to-Consumer Bone-Conduction Device: A Multiple Methods Intervention Study

**DOI:** 10.2196/66013

**Published:** 2025-07-15

**Authors:** Thomas Hampton, Mark Fletcher, Alan Sanderson, Manuel Loureiro, Kevin Mortimer, Mahmood F Bhutta

**Affiliations:** 1 Department of Clinical Sciences, Liverpool School of Tropical Medicine, Pembroke Place, 160 Mount Pleasant, Liverpool, L3 5QA, United Kingdom, +44 7805098711; 2 Institute of Life Course and Medical Sciences, University of Liverpool, William Henry Duncan Building, 6 West Derby Street, Liverpool, L7 8TX, Liverpool, United Kingdom, 1 0151 794 2064; 3 University Hospitals Sussex NHS Foundation Trust, Brighton, United Kingdom; 4 Malawi-Liverpool-Wellcome Trust Clinical Research Programme, Chipatala Avenue PO Box 30096 Chichiri, Blantyre, Blantyre, Malawi, 1 +265 (0)9929712; 5 The Institute of Sound and Vibration Research, University of Southampton, Southampton, United Kingdom; 6 Brighton and Sussex Medical School, Brighton, United Kingdom; 7 Cambridge Africa, University of Cambridge, Cambridge, United Kingdom; 8 Department of Paediatrics and Child Health, School of Clinical Medicine, College of Health Sciences, University of KwaZulu Natal, Durban, South Africa; 9 Department of Respiratory Medicine, Liverpool University Hospitals NHS Foundation Trust, Liverpool, United Kingdom

**Keywords:** hearing loss, auditory perception, wearable electronic devices, medical device, health resources, rehabilitation, cost efficient, mixed-methods study, speech recognition, hearing aids, rehabilitation technology, rehabilitation device, assistive technology, otitis media, health intervention

## Abstract

**Background:**

Hearing loss affects 20% of the global population, including 250 million experiencing chronic suppurative otitis media, which can present challenges for conventional hearing aids due to ear discharge. Although assistive technology for hearing is available in high-income settings, provision is poor in low-income settings due to high costs and low availability of audiology services, reaching approximately 3% of those who could benefit from it.

**Objective:**

This study aimed to evaluate the performance of a low-cost self-fitted direct-to-consumer bone-conduction headset for individuals with conductive or mixed hearing loss.

**Methods:**

We conducted a multiple methods study to test the efficacy and acceptability of this device using a purposive sample. Participants with a range of conductive and mixed hearing loss underwent speech-in-quiet speech audiometry with and without the device and took part in feedback interviews exploring their subjective impressions of the device.

**Results:**

In 33 participants, the device improved speech recognition in those with bone conduction thresholds <50 dB by a median of 11%, with larger air-bone gap associated with larger improvement. Participants rated the device positively on weight, style, and ease of use.

**Conclusions:**

This multiple methods study assessed the acceptability and efficacy of a low cost self-fitted bone-conduction device in adults. We found the device provides hearing benefit for those with conductive or mixed hearing loss (with bone conduction thresholds <50dB HL). Those with significant conductive hearing loss were measured to have their speech perception significantly improved. Participants had a mixed response to device aesthetics. Further studies should seek to establish if this type of device has effectiveness in real-world trials and which individuals are most likely to benefit. This low cost device could provide hearing benefits to millions of people without access to other devices. Product designers and clinical researchers should explore device optimization. Given the economic impacts of hearing loss across the globe, this style of self-fitted device could represent a paradigm shift in future assistive technology for hearing loss, in both high and low resource settings.

## Introduction

Hearing loss affects 1.5 billion people globally with an estimated 250 million people with chronic suppurative otitis media (CSOM) [1-3], which carries the additional stigmatizing burden of a discharging ear (otorrhea) and associated difficulty using conventional in-ear air conduction hearing aids [4]. In children, hearing loss impairs cognitive development, language, social-emotional skills [5] and other milestones [6]. In low-resource settings, the burden of hearing loss is further compounded by poor health provision and infrastructure to address the needs of its hearing-impaired population [7]. One estimate found >400 million people are “in need” of hearing aids and called for the development of innovative low-cost technologies and effective service delivery models [8].

In UK primary schools in 2019, only 43% of children with any level of hearing loss reached expected numeracy and literacy (vs 65% of typically hearing pupils), and by age 16 years, children are on average 17.5 months behind the attainment of their typically hearing peers [9]. This impacts throughout the life course, as adult employment rates for those with hearing loss are 65%, compared to 79% in people with no long-term health issue or disability [10]. In England, 48% of people aged 50 years and older have some degree of hearing loss [11], and this costs the United Kingdom £25 billion (US $33 billion) a year in lost productivity and unemployment [12]. Efforts to address hearing loss are necessary: medically, socially, and economically, across the world.

Hearing loss can be conductive, sensorineural, or mixed, and many mild, moderate, and severe types of hearing loss of all causes can be rehabilitated with conventional air conduction hearing aids (which transmit sound via the air to the tympanic membrane). However, for patients with conductive or mixed hearing loss (eg, due to CSOM), surgical intervention or bone conduction devices (BCDs), which transmit sound via the skull to the cochlea, are an alternative for rehabilitation [3]. In cases where the conductive component of hearing is large (air-bone gap>25‐35 dB), some users demonstrate better [13,14] or comparable [15] word identification using a BCD compared to air conduction hearing aids. At present, bone conduction is used in only a minority of cases of hearing loss. For example, in the National Health Service (NHS) in England, around 350,000 new air-conduction hearing aids are provided each year (Prof K Munro, personal communication), in comparison to 1292 BCDs surgically implanted between 2022 and 2023 [16].

Clinically fitted BCDs may be external (nonimplanted, eg, attached via headbands) or implanted into the skull. If not implanted, their audiological function is suboptimal as the headband pressure and depth of skin overlying the skull will attenuate their performance [17]. Hence, the normal clinical pathway is that after successful trial of an external device, a BCD is surgically implanted into the skull and is usually highly effective [18,19]. Such devices are classed and regulated as a medical device and are costly, with recent low estimates of £3150 (US $10,000 to US $17,000; excluding costs of the surgical procedure) [20].

The high cost of implanted devices and the need for surgery means that BCDs are likely underused [21]. External BCDs could allow this technology to be expanded, for example, to treat the most common cause of pediatric hearing loss, glue ear (chronic otitis media with effusion), as this causes a conductive hearing loss that is temporary [22], where implantation would not be appropriate. It could also be used to aurally rehabilitate adults and children with CSOM, a disease most prevalent in low- and middle-income countries [2], where access to traditional in-ear air conduction aids is sparse and often inappropriate, as they are less effective in the presence of otorrhea and may exacerbate it [23]. However, medically certified external BCDs (typically fitted attached to a soft headband) are of comparable cost to implanted devices.

**Figure 1.**
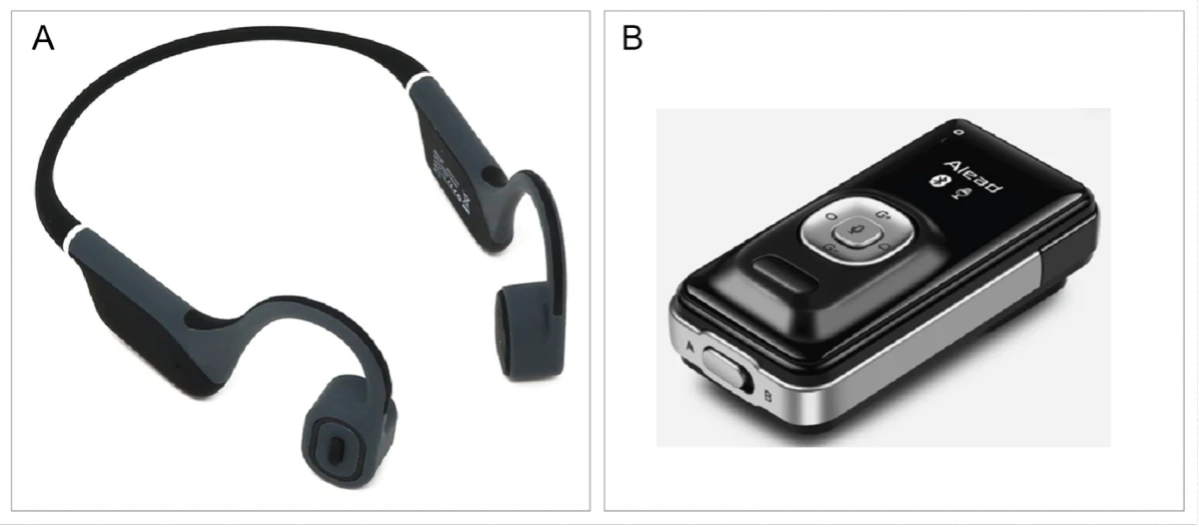
(A) Raspberry Pi Hear Glue Ear Headset (SKU: 105890) [24]. (B) Alead Nolan LiveMIC2 Bluetooth Wireless Microphone [27].

Previous pilot studies have shown benefit from a low-cost direct-to-consumer external bone conduction headset supplied by "Raspberry Pi Foundation” (Cambridge, United Kingdom) for rehabilitation of children with glue ear [24]. In 2 small trials, this device paired with a Bluetooth microphone (see Figure 1) improved word identification score by 23% in 20 children with hearing within normal limits [25] and reduced word discrimination threshold of distant speech in both quiet and in noise for 19 children with otitis media with effusion [26]. The majority of parents (22/26) opined that this device consistently helped their child to hear [24].

**Figure 2.**
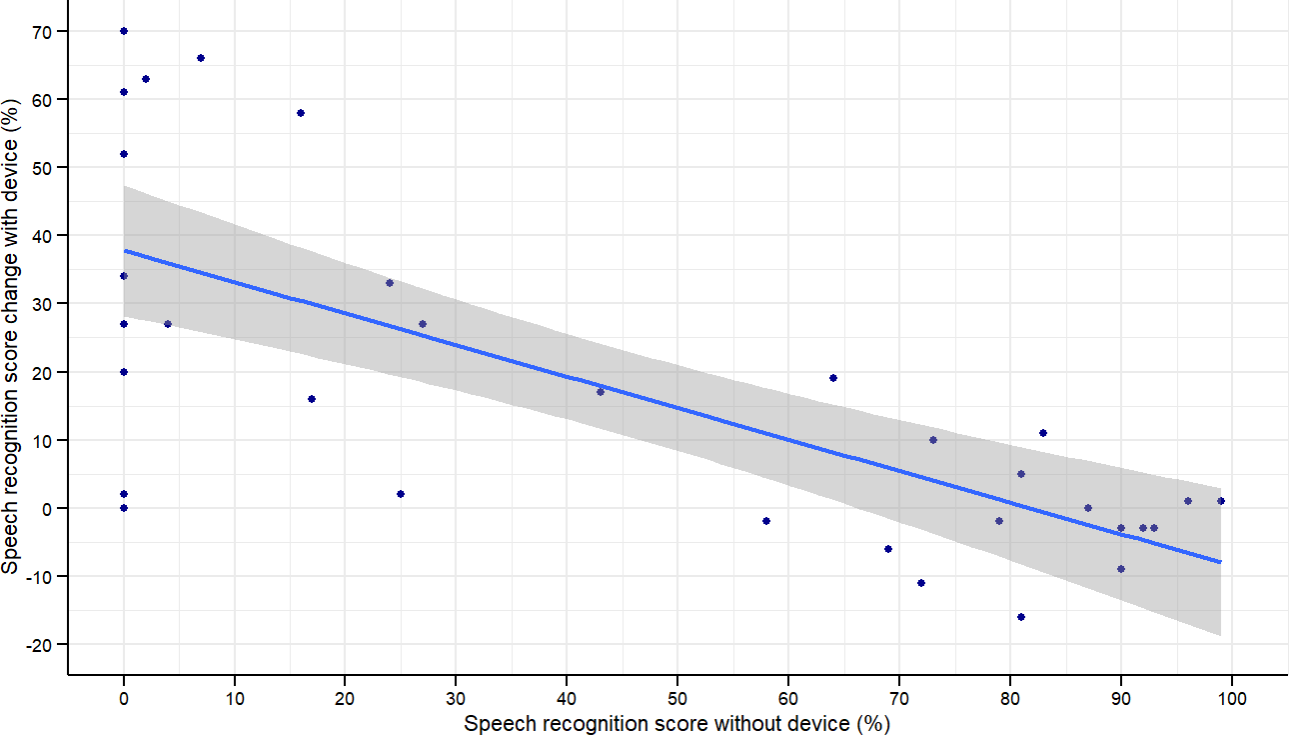
Plot of percentage (%) speech recognition score change with device and baseline percentage (%) score without device.

In this multiple methods study, we tested the efficacy of this device for speech recognition in quiet listening conditions in adults and to assess participant acceptance of the device. We purposively recruited adult patients with conductive and mixed hearing loss. The inclusion of mixed hearing loss allowed us to measure the effect of raised bone conduction hearing threshold on word identification, which is important for describing the fitting range of the device for future applications. We hypothesized that the device would markedly improve speech performance on average across participants. We further hypothesized that unaided speech performance (without the device) would predict the amount of benefit, with people who are high baseline performance benefiting less than those who are low baseline performance. We expected this effect to be most pronounced where the bone conduction thresholds tended towards normal limits and were combined with a large air-bone gap.

## Methods

### Ethical Considerations

Ethical approval and sponsorship for the study was obtained from the Liverpool School of Tropical Medicine (21-079) and the national NHS Health Research Authority Research Ethics Committee (IRAS 304976). The study was registered with the University Hospitals Sussex Clinical Research Department. All participants gave informed consent to participate.

Although patients and the public were not directly involved in the development of the research question, design and conduct of this study, results will be disseminated to study participants either by post or email as per their expressed preference.

### Participants

Purposive sampling sought eligible adults aged ≥18 years with documented conductive or mixed hearing loss regardless of etiology (unless otherwise indicated in exclusion criteria), including those previously fitted with hearing aids or BCDs (externally worn or surgically implanted). We excluded participants unable to converse in English (English did not need to be first language, but participant-determined fluency was the criterion), children, those with cognitive impairment that would limit word identification or device engagement, and those with medical conditions that would preclude wearing the device (such as severe eczema).

To recruit our sample, we interrogated our database of individuals who have previously attended outpatient ear, nose, and throat surgery or audiology clinic at University Hospitals Sussex NHS Foundation Trust, and recruited participants with a range of average bone conduction hearing levels. Details regarding previous aiding and rehabilitation are provided in Multimedia Appendix 1. We created *a* priori subgroups based on the World Health Organization (WHO) hearing-impairment grading system [7], but adapted to bone conduction thresholds only, to define mild (21‐34 dB HL [decibels Hearing Level] mean bone conduction threshold), moderate (35‐49 dB HL), and severe to profound (>50 dB HL) hearing loss [28,29].

Hearing threshold levels (HTLs) were measured using pure tone audiometry according to ISO [International Organization for Standardization]:8253‐1 following the British Society of Audiology (BSA) recommended procedures (2018) in a soundproof booth (Eckel Audiology Suites, acoustic compliance HTM [UK Health Technical Memorandum] 2045/ISO 8253). Air conduction hearing thresholds were measured at 250, 500, 1000, 2000, 4000, and 8000 Hz for each ear independently. Bone conduction hearing thresholds were measured at 500, 1000, and 2000 Hz. Masking was applied where indicated, again as per BSA recommended procedures (2018). Hearing thresholds were measured using an audiometer (MedRx Avant A2D+ Audiometer, MedRx Inc) fitted with Telephonics TDH-39 supra-aural air-conduction transducers, 3M E-A-RTONE 5A insert earphone transducers, and Radioear B-81 bone-conduction transducer.

### Measuring Speech Recognition Score

The BCD was a prototype (model G18) produced in collaboration by Hear Glue Ear and Raspberry Pi [24], paired via Bluetooth with an Alead LiveMIC2 Bluetooth Wireless Microphone (model A2DP; Alead Inc).

The efficacy of the BCD was measured by comparing word identification as a surrogate for speech recognition scores (SRS) from unaided (BCD inactive) and aided (BCD active) conditions. We used Arthur Boothroyd AB word lists comprising 15 lists of 10 monosyllabic, isophonemic consonant–vowel–consonant words [30]. We used AB testing because it is considered reliable (using phonemic rather than whole-word scoring), because normative data exist for adults and children [31], and because it is potentially superior in the assessment of speech audibility compared to Bamford, Kowal, Bench testing because context cues are not available and responses are less predictable [32].

The prerecorded English accent AB word lists were administered via a MOTU M4 Audio interface (MOTU, Inc) running a bespoke MATLAB program (MATLAB version R2022a) to present AB word lists in English (once with BCD active, once with BCD inactive). The air conduction dial level was set to give speech output level of 70 dBA root mean square through Etymotic ER3 insert earphones (Etymotic Research). Speech material presentation was diotic in all conditions (with both ears receiving the same signal). Insert earphones were calibrated using a Bruel and Kjaer ear simulator (Artificial Mastoid Type 4930 and Bruel & Kjaer Type 2250 sound level meter ISO 389‐2). The bone conduction headset output level was set to give speech output level of 70 dB re 1 µN (with no frequency weighting), calibrated using a Bruel and Kjaer Type 4930 artificial mastoid. The air conduction speech stimulus was presented diotically at 70 dBA root-mean-square in both aided and unaided conditions.

For each condition, participants underwent a practice 10-word list followed by 5 test lists each formed of 10 consonant-vowel-consonant words presented in random order (uniform distribution). Responses were scored as the proportion of phonemes repeated correctly in each list of words (3 phonemes per word) [33].

We compared phoneme SRS from unaided and aided conditions. We recorded the number of participants in each hearing severity subgroup who attained a 50% aided word identification score (our definition of functional hearing) [34]. Statistical analysis was conducted in RStudio (Posit, 2020).

### Questionnaire and Feedback on Device Performance and Acceptability

In total, 32 participants took part in semistructured feedback, of mean duration 8 (range 3‐16) minutes. The feedback reported here focused on perceptions and impressions of the new device.

Each participant was invited to provide their opinion on device acceptability and performance using a 5-point Likert scale questionnaire. Extreme score anchors of heavy-light, ugly-stylish, and difficult-simple were used in the weight, style, and ease-of-use questions respectively.

Participants were then invited to provide verbal feedback to record more detail of their opinion. A pilot interview was used to adjust schedule prompts before finalizing, with an aim to interview for approximately 10 minutes. Interviews were undertaken in an audiology clinic room at the study site on the same day as the main experiment. An information sheet on the verbal feedback was provided via email before and offered again on the day. An audio recording of the interview was made (Philips 3200 SpeechMike Pro USB Microphone LFH3200) and anonymously mapped to study ID using Zoom software (Zoom Video Communications). Anonymized handwritten field notes were also taken.

Audio recordings were transcribed [35], coded using NVivo V.12 Pro software (Lumivero), and content analyzed for associations with audiometric and Likert scoring as well as for additional insights about design and acceptability. An inductive reflexive thematic analysis approach [36] was used to develop broader themes to illustrate participant experiences and selected excerpts used to summarize results. Due to the first author’s epistemological standpoint about interview data, no frequentist attempts at triangulation, reproducibility, or mitigation of bias were made for the qualitative elements of this study.

## Results

### Participants

A total of 167 participants were contacted by letter, 36 expressed interest, and 33 attended and completed the study. Ages of participants ranged from 21 to 82 with a median of 70 (mean 66) years. Self-reported genders were male (n=15), female (n=16), and bigender or “any” (n=2). Participants were categorized according to average bone conduction HTL: 19 in mild (>20‐34 dB HL), 10 in moderate (35‐49 dB HL) and 4 in severe (>50 dB HL). The conductive element of hearing loss, the “air-bone gap,” ranged from 4 dB to 59 dB (median 27 dB).

### Audiometric Outcomes

**Figure 3.**
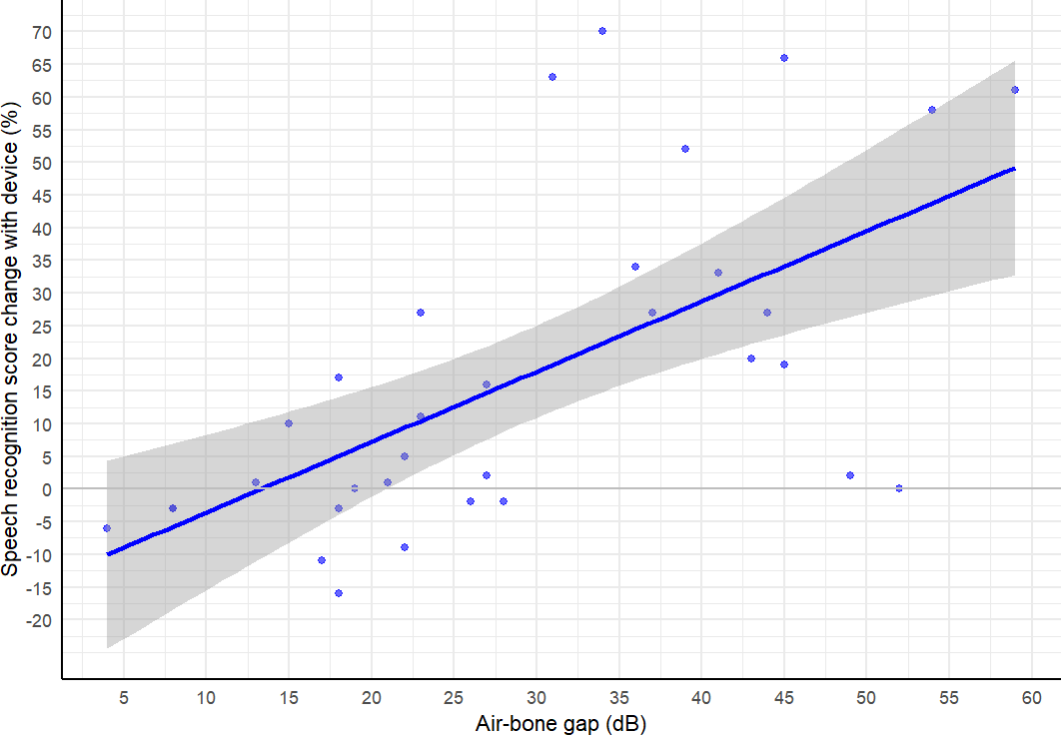
Improvement in % speech recognition score with device, depending on participant air bone gap in decibels (dB).

Median speech recognition score change with the device across all 33 participants was 10 (IQR 29). Figure 3 shows the relationship between unaided SRS, which represents baseline performance with no BCD benefit, nor lip reading, and SRS change between unaided and aided conditions. Participants in this cohort with lower unaided SRSs exhibit a positive trend toward greater aided score changes. The *R*^2^ of the regression model is 0.52 (*P*<.01), regression coefficient (β) was −0.463 (SE 0.08), and *t*-statistic −5.74.

**Figure 4.**
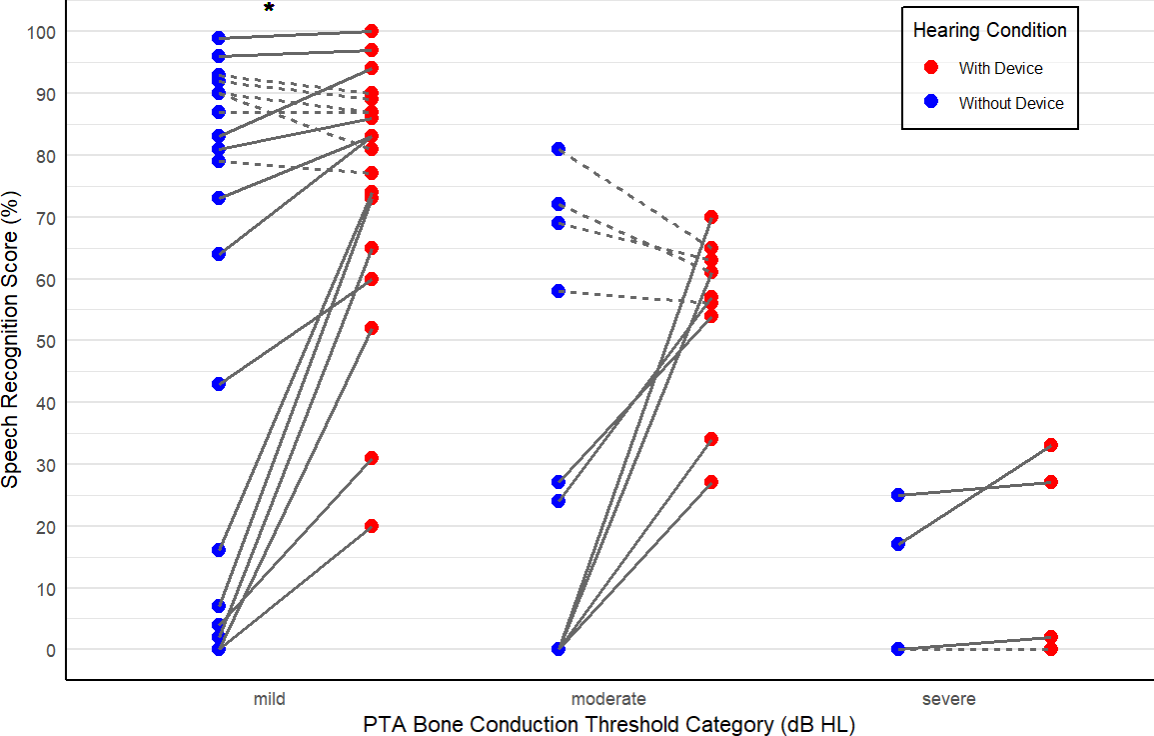
Scatterplot of speech recognition scores with (aided) and without device (unaided) for each participant. Solid line indicates an increase in score with device, and dashed line indicates no change or worse. The asterisk indicates statistically significant (*P*<.01) improvement with device for that group.

**Figure 5.**
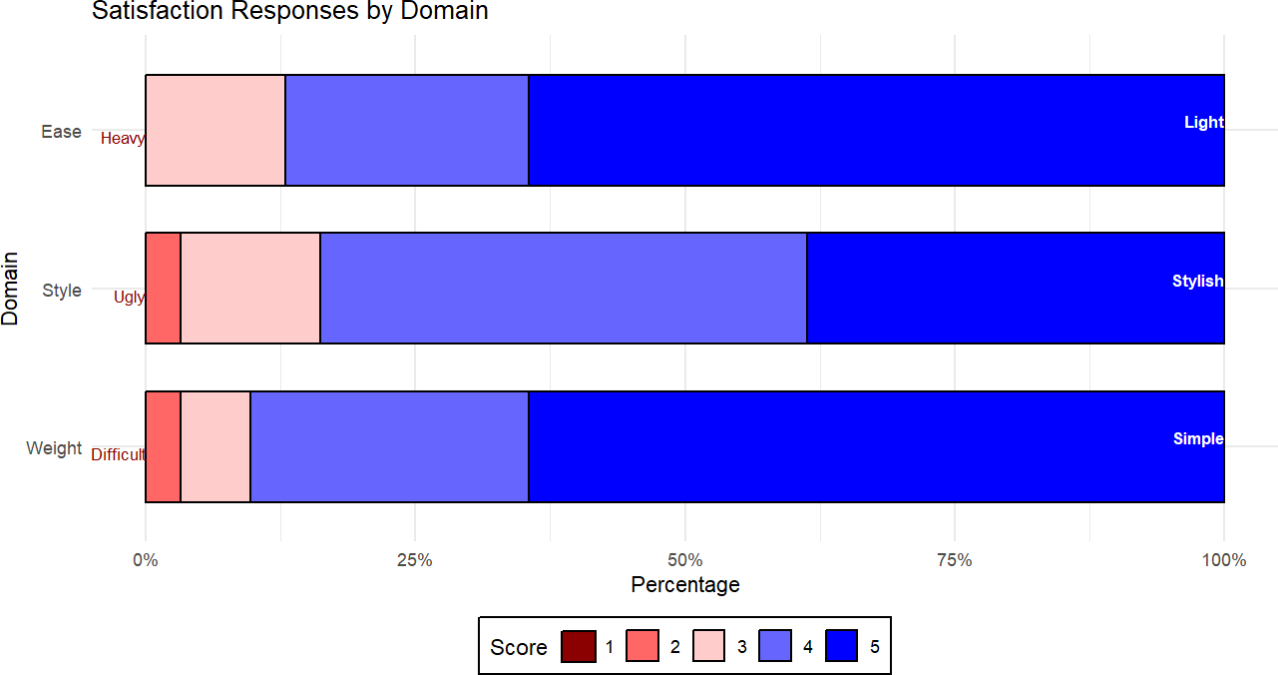
Histogram of device satisfaction score responses from 31 participants. Higher scores were taken to be “positive” impressions of the device, based on discussion with participants to clarify directionality even with anchors.

We next investigated possible mechanisms to explain the relationship between unaided and aided SRS. We used a hypothesis-driven approach with forward selection of variables in linear regression models for change in score, optimizing Akaike Information Criterion with each iteration [37]. In total, 9 regression models were fitted, and we found the model with the best Akaike Information Criterion (291) included only the variables of air-bone gap (Figure 4) and category of bone conduction HTL (Figure 5).

Figure 4 shows the relationship between air-bone gap, which represents the magnitude of hearing loss that is conductive, and the SRS change. Because the device provides sound transmission via bone conduction, we expected that greater magnitude of conductive hearing loss (air-bone gap) would be associated with greater aided SRS change. Regression analysis confirmed this prediction, with an adjusted *R*^2^=0.459 (suggesting approximately 46% of variance in score could be explained by the model), and a coefficient of 1.2 (suggesting that for every 1 dB increased air bone gap, the AB score increased by >1 when using the device, *P*<.001).

Next, we investigated the effect of bone conduction HTL, which represents the magnitude of underlying sensorineural hearing loss (SNHL). Participants were categorized using the WHO hearing-impairment grading system according to their average bone conduction HTL. Figure 5 shows unaided (blue points) to aided (red points) SRS change for participants in the mild, moderate, and severe categories of bone conduction thresholds. Comparison of means between unaided and aided conditions across all conditions found a significant improvement in SRS (pooled Wilcoxon signed-rank test, V=409, Z=2.30, *P*<.01). Subgroup analysis showed a difference in SRS between unaided and aided conditions in the mild category (Figure 3, Wilcoxon signed-rank test, V=145, Z=2.01 *P*=.01, or *P*=.03 with Bonferroni correction for 3 comparisons), but differences in the other groups did not reach significance (z=1.78, uncorrected *P*=.08 for moderate and z=0.37, uncorrected *P*=.17 for severe).

The median change in SRS for each bone conduction hearing threshold category is presented in Table 1. Using pooled data, participants with hearing thresholds <50 dB (mild and moderate categories) showed median gain in SRS of 11%.

Of 33 participants, 17 had worse than 50% unaided SRS, of which 9 participants improved to >50% (in these 9, median SRS went from 7 unaided to 61 with device).

**Table 1.** Change in AB speech recognition score (%) by pure-tone audiometry bone conduction threshold category.

Bone conduction threshold category	Median change in aided versus unaided SRS^a^	IQR of change
Mild	+10	−1.0 to 23.5
Moderate	+27	−5.0 to 33.8
Severe	+2	1.5 to 5.5

a SRS: speech recognition scores.

### Feedback on Device Performance and Acceptability

Two participants did not fully complete the questionnaire, and for analysis, their scores were excluded. For the remaining 31 participants, mean scores (from 1 to 5) for weight, style, and ease of use were 4.5 (SD 0.74), 4.1 (SD 0.80), and 4.6 (SD 0.69), respectively. No individuals gave a score of 1 (worst score), and the ease of use and appearance was scored 2 for one participant respectively (refer to Figure 6). We found no clear relationship between SRS and verbal reported perceptions of the device. Although there was some negative feedback from participants with poor SRS about how well the device performed (particularly among the severe hearing loss group), some of the lowest (even negative scoring) SRS participants felt the device “worked,” “worked well,” or was “better than… (their current) device.”

We also found no clear relationship between normalized Likert scale satisfaction scores and verbal reported perceptions of the device. Participants with maximum satisfaction scores still had recommendations for improvements or preferred a discrete device, and one said outright “I didn’t like the look of it.” Conversely, where participants gave poor satisfaction scores, occasionally weight, style, and ease of use were still described favorably, with one low-scoring participant reporting “a good design and a nice weight… you can’t feel that you’ve got it on, really.”

We asked participants if they would recommend the device to friends and family if they had a similar hearing impairment. In total, 79% (26/33) of participants said they would recommend.

### Themes Developed From Verbal Feedback

Many participants described their adoption of new hearing technology or devices as “transforming their life” and their quality of life without the aids as significantly impaired.

*If I didn’t have my hearing aids then I’d be lost completely.*

[Participant 12]

Regarding the new device specifically, participants were divided about aesthetics. There were no obvious respondent characteristics to identify who liked or did not like the appearance, but a larger sample size could reveal trends. Responses largely correlated to personal preference about the appearance of the device (namely whether aids should be obvious and celebrated or unobtrusive and remain discrete). Some participants approved of the fact that the device (given its original purpose) looked like a music player or headphones.

People would wear them because they look like normal headphones… My cousin has some of these that he just uses for music!

[Participant 2]

They looked cool, and you wouldn’t be embarrassed using them, because a lot of people go around with earphones anyway.

[Participant 4]

Several participants alluded to appearance, and perceptions of disability and attitudes in society. Some participants were happy the new device masked their disability, as it looked like a music device, but others liked that it proudly drew attention to their condition:

I mean if you’re going to be deaf you’re going to be deaf, so you might just as well flag it up.

[Participant 3]

If it was smaller, I could hide it under my hair…Because I’m quite vain. (Laughter).

[Participant 6]

For many participants, there were reflections on normality and disability, the hidden nature of deafness, and the difficulty experienced by participants coping with a hidden disability in an intolerant and ableist society. Many reflected on personal shame or stigma.

For some participants, having an obvious hearing aid of any description was something they found troubling given previous experiences. Many participants said they were more comfortable with devices as they grew older, reflecting perhaps that changes from their initial objections (which at the time, most put down to aesthetics) might represent growing personal acceptance of their hearing disability.

## Discussion

### Principal Findings

We investigated the efficacy of a low-cost direct-to-consumer BCD to improve SRS in participants with conductive and mixed hearing loss. The main findings were that (as would be anticipated) participants in this study with lower unaided SRS showed a trend toward the greatest improvement in aided SRS. Participants with larger air-bone gaps also showed a greater improvement in SRS (approximately 1% per dB of gap). The mean change in SRS for each category of bone conduction threshold severity was 17.4 for mild (SD 10), 21.7 for moderate (SD 27), and 5 for severe (SD 2), which was statistically significant only for the mild category group.

Our findings suggest that this low-cost device is likely to benefit individuals with a conductive hearing loss where underlying sensorineural thresholds are largely preserved, and, consistent with previous research [13,14], that benefit is more likely where air-bone gap is large.

Although statistical significance was not reached for the moderate bone conduction threshold group, 4 of 10 participants showed SRS change greater than 20%. This variability in outcome has several possible explanations. To categorize participants, we used average bone conduction HTLs, so it is possible that different shapes of bone conduction audiograms are better suited to the frequency response characteristics of the device. Interindividual variability in the moderate group could also be affected by characteristics other than bone conduction HTL, such as cognition [38,39] or capacity for speech recognition using additional information from lip cues and context [40]. In the severe bone conduction threshold group, there was no evidence of benefit (although this contained only four participants), which may be due to the reasons above, but more likely reflects the fact that this BCD is unable to rehabilitate when there is underlying severe SNHL.

Of 33 participants, 22 had an improved score, and nine had a worse score with the device. Of these, 5 participants with worse scores and 5 with better scores had scores equal to or within 5% of their original score. Concerns about test-retest within participant speech score variability are well documented [41]. Reassuringly, if all 10 participants with these small, potentially nonsignificant score changes were excluded from analysis, then the median score change remains significant to *P*<.05 for the mild but not the moderate threshold group, leaving our overall conclusions unchanged. Future studies should seek to predetermine minimally significant change levels. A score of 50% or greater on the Arthur Boothroyd word test has previously been defined as a marker of satisfactory rehabilitation with cochlear implants [42]. In our cohort, amongst the 16 participants where SRS was worse than 50% without the device, 9 achieved greater than 50% with the device, and mean SRS improvement in this group was 34 (SD 24).

Our qualitative investigation showed favorable opinions towards the size, weight, and ease of use of the BCD, with normalized mean satisfaction from these three variables of 0.8. Our interview data found that both word identification and questionnaire scores are imperfect surrogates of participant satisfaction. In the future, the satisfaction scores used for this questionnaire may benefit from correlation with an overarching construct of device satisfaction or a hearing-specific questionnaire.

Our findings suggest potential for the use of this kind of device. First, and perhaps foremost, the device could provide options for low-cost rehabilitation for those with conductive or mixed hearing loss in low-resource settings. In particular, many cases of hearing loss due to CSOM could be rehabilitated with a BCD [3]. A low-cost direct-to-consumer approach circumvents issues of conventional approaches to hearing aid provision in low-resource settings, including the need to travel to and attend a health facility, a lack of audiologists in such facilities (for example found to be 0.8 audiologists per million population in sub-Saharan Africa [43]), and the costs of a hearing device (an air conduction hearing aid can cost up to US $6000 in high-income settings [44], but commercially available BCDs for sports and recreation are available for £160 (US $149) [45], and the device used in this study was purchased for £18 (US $24.59) [24] (with the Bluetooth microphone an additional £79 [US $107.92]) [27]. These factors contribute to the fact that as few as 3% of people in low-income settings who would benefit from a conventional hearing aid currently have access [44], even though this is where an estimated 90% of people with disabling hearing loss live [46]. A bone conduction externally worn device could be reused or redistributed as it would not need a personalized mold or programming and might have less likelihood of being a vector for microbial transmission compared to in ear air conduction hearing aids [47]. A low-cost device could be made available through conventional retail outlets or schools, demedicalizing hearing rehabilitation and aligning with the WHO promotion of community ear and hearing care [48].

Other causes of conductive or mixed hearing loss could also be rehabilitated by this device, both in low- and high-resource settings. Glue ear (otitis media with effusion) is the most common cause of childhood hearing loss, but usually resolves, making it highly appropriate for rehabilitation with a nonimplanted BCD, and potentially avoiding the need for surgery (with preliminary evidence to support use for this purpose from the studies by Holland Brown [24,25,49]). However, this needs further investigation as a previous datalogging study from the United States [50] found that as many as 73% of children using an external BCD had low use (mean of 1.25 h a day).

Research exploring the pathways for bone conduction sound transmission has been conducted by the Stenfelt laboratory, showing that cochlear excitation is produced through 5 different routes. This evidence shows the profile of transmission for mastoid transducer placement includes all 5 but is dominated by cochlear fluid inertia [51,52]. In contrast, a recently published study from the same group shows the route of transmission for the headset investigated here is dominated by ear canal sound pressure because of the preauricular transducer position [53]. This transmission route depends on the oscillation of the soft tissue of the external ear canal, which by being coupled to the air in the canal produces transmission like air conduction. Therefore, conductive hearing losses caused by outer- and/or middle-ear abnormality will reduce both air and bone conduction transmission from this headset. An extreme example such as aural atresia may significantly reduce the effectiveness of headsets with preauricular positioned transducers. The investigation presented here included a range of types and severity of conductive hearing loss. Although none had atresia or microtia, it is possible the anatomical variations could have different effects on the ear canal sound pressure route of transmission. This may have contributed to the heterogeneity observed in the word recognition scores.

### Study Limitations

The aim of our study was to measure the effect of the BCD with a controlled input level speech stimulus via air conduction and bone conduction (aided) and air conduction only (unaided). This was a repeated measures within-subject design with randomized order of (aided or unaided) conditions, but it is possible that participants could feel the difference between conditions because of the vibrotactile sensation associated with bone conduction.

The device was evaluated in a quiet listening environment with microphone input speech clear and stable, which may not translate to less favorable listening environments.

Some of the variability in outcomes within bone conduction threshold groups may relate to asymmetry in hearing threshold between ears, which can decrease binaural summation [54], although SRS is usually best predicted by “better” ear performance on bone conduction thresholds [55]. Outcomes of cochlear implantation have been shown to better map to total auditory receptivity of the brain (what signal, if any, was received) as opposed to ear-specific events [56]. Future work and larger sample sizes may allow us to investigate at what threshold of bone conduction of the worse ear this device may improve SRS and whether this correlates to the worse ear gaining audibility, the effects of binaural summation, or (as it seems) predominantly air-bone gap.

The median age in our cohort was 70, and so a large proportion of our cohort will have presbycusis [57], with associated limitations to speech recognition performance [58]; a younger cohort may achieve greater benefit, and it may be that device performance would differ in those with SNHL versus from other etiology.

We did not assess previous rehabilitation (namely previous experience, duration, and quality with other assistive devices), which may correlate with capacity to benefit and word identification ability. Future studies could seek to record the duration of previous auditory deprivation in addition to prior device use, as this could impact participants’ baseline auditory processing, and cognitive ability may impact the capacity to benefit from the device.

We used bespoke approaches to qualitative assessment and are aware that other questionnaires to evaluate assistive hearing technology have been validated [59,60]. Participants did not adjust settings or handle the device during the word identification task, and so our qualitative findings are based on the participant having only a brief interaction with the device.

### Future Work and Wider Applications

This multiple methods study demonstrates efficacy for a low-cost self-fitted, commercially available BCD to improve SRS in individuals with conductive or mixed hearing loss.

Objective measures of device output in laboratory conditions and normative head tension and performance of individuals with typical hearing would help interpret existing and future investigations of speech performance using the device. This could guide engineering optimizations of the device such as headband tension, signal processing, and transducer characteristics. Exploring opportunities to optimize costs versus performance could include potentially differentiated offerings for use in low and high-resource settings. Future studies should also explore the benefit of this BCD in different clinical scenarios, for example, for CSOM or glue ear.

In summary, this study shows that a low-cost direct-to-consumer BCD effectively improved word identification, had high user acceptability, and could support a shift toward self-fitted (nonmedical) rehabilitation for conductive or mixed hearing loss, in both high- and low-resource settings.

## Supplementary material

10.2196/66013 Multimedia Appendix 1 Table of prior hearing rehabilitation details (ACHA: air conduction hearing aid).

## Abbreviations

BCD: bone conduction devices
BSA: British Society of Audiology
CSOM: chronic suppurative otitis media
dB HL: decibels Hearing Level
HTL: hearing threshold level
HTM: UK Health Technical Memorandum
ISO: International Organization for Standardization
NHS: National Health Service
SNHL: sensorineural hearing loss
SRS: speech recognition scores
WHO: World Health Organization
