# Supplementary material for: Improved Speech Recognition in Adults With Conductive or Mixed Hearing Loss Using a Direct-to-Consumer Bone-Conduction Device: A Multiple Methods Intervention Study
Source: JMIR Rehabil Assist Technol. 2025 Jul 15;12:e66013. doi: 10.2196/66013 (PMC12282675; doi:10.2196/66013)
Supplement: Multimedia Appendix 1 [file rehab-v12-e66013-s001.docx]

Appendix

| **Attribute** | **n** |
| --- | --- |
| Side |  |
| Left | 10 |
| Right | 14 |
| Bilateral | 9 |
| Prior device |  |
| ACHA | 4 |
| BAHA 5 SP | 1 |
| BiCROS | 2 |
| Ponto 3 | 1 |
| Ponto 3 SP | 15 |
| Ponto 4 | 6 |
| Ponto Plus Power | 2 |
| Ponto Pro | 1 |
| Ponto Pro Power | 1 |
| Years of prior device use |  |
| 2-3 years | 4 |
| 4-5 years | 4 |
| 6-7 years | 9 |
| 8-9 years | 6 |
| 10-11 years | 4 |
| 12-15 years | 3 |
| 16+ years | 3 |
| Current transcutaneous users | 4 |

Appendix table of Prior Hearing Rehabilitation details (ACHA: air conduction hearing aid)
